# *Artemisia halodendron* Litters Have Strong Negative Allelopathic Effects on Earlier Successional Plants in a Semi-Arid Sandy Dune Region in China

**DOI:** 10.3389/fpls.2020.00961

**Published:** 2020-06-25

**Authors:** Yongqing Luo, Zhong Du, Zhiqiang Yan, Xueyong Zhao, Yuqiang Li, Haibo Jiang, Yue Yang, Mai-He Li

**Affiliations:** ^1^ Naiman Desertification Research Station, Northwest Institute of Eco-Environment and Resources, Chinese Academy of Sciences, Lanzhou, China; ^2^ School of Land and Resources, China West Normal University, Nanchong, China; ^3^ Forest Dynamics, Swiss Federal Institute for Forest, Snow and Landscape Research WSL, Birmensdorf, Switzerland; ^4^ School of Environmental and Municipal Engineering, Lanzhou Jiaotong University, Lanzhou, China; ^5^ Urat Desert-Grassland Research Station, Northwest Institute of Eco-Environment and Resources, Chinese Academy of Sciences, Lanzhou, China; ^6^ Chengdu Institute of Biology, Chinese Academy of Sciences, Chengdu, China; ^7^ Key Laboratory of Geographical Processes and Ecological Security in Changbai Mountains, Ministry of Education, School of Geographical Sciences, Northeast Normal University, Changchun, China

**Keywords:** allelopathic index, litter extracts, plant succession, seed germination, vegetation restoration

## Abstract

*Artemisia halodendron* Turcz. ex Besser occurs following the appearance of a pioneer species, *Agriophyllum squarrosum* (L.) Moq., with the former replacing the latter during the naturally vegetation succession in sandy dune regions in China. A previous study revealed that the foliage litter of *A. halodendron* had strong negative allelopathic effects on germination of the soil seed bank and on the seedling growth. However, whether this allelopathic effect varies with litter types and with the identity of plant species has not yet been studied. We, therefore, carried out a seed germination experiment to determine the allelopathic effects of three ltter types of *A. halodendron* (roots, foliage, and stems) on seed germination of six plant species that progressively occur along a successional gradient in the semi-arid grasslands in the Horqin Sandy Land of northeastern China. In line with our expectation, we found that the early-successional species rather than the late-successional species were negatively affected by *A. halodendron* and that the allelopathic effects on seed germination increase with increasing concentration of litter extracts, irrespective of litter types. Our study evidenced the negative allelopathic effects of *A. halodendron* on the species replacement and on the community composition during dune stabilization in the Horqin Sandy Land. Further studies are needed to better understand the successional process and thus to promote the vegetation restoration in that sandy dune region as *A. halodendron* itself disappeared also during the process.

## Introduction

Plant litter can affect the structure and composition of plant communities directly through its influence on the emergence and early growth of seedlings (Sayer, 2006; Li K. et al., 2016). The influence of plant litter on plant establishment can be negative or positive, and these influences differ among species, litter types, and litter quantity (Hovstad and Ohlson, 2008). Litter affects plant establishment not only by modifying the microenvironment such as changes in the light supply (Weltzin et al., 2010), soil moisture (Wolkovich et al., 2009), soil nutrient content (Fahnestock et al., 2000), and herbivores (Reader, 1993; Facelli, 1994), but also by means of allelopathy derived from the release or leaching of allelochemicals during litter decomposition (Cheema et al., 2013; Loydi et al., 2015). Allelopathy is defined as the stimulatory or inhibitory effects of one plant upon another, including interactions with microorganisms (Rice, 1984). For example, the foliage litter of *Artemisia halodendron* can affect the establishment of other species by allelopathy in degraded sandy grassland (Luo et al., 2017). Root litter of *Eucalyptus urophylla* in a subtropical forest ecosystem significantly inhibited the germination and height growth of *Schima superba*, *Michelia macclurei*, and *Elaeocarpus sylvestris* due to allelopathy (Zhang and Fu, 2009).

Allelopathy is an important phenomenon that plays a significant role in plant dominance, succession, and the formation of plant communities (Chou, 2006). During allelopathy, a plant releases phytotoxic compounds (allelochemicals) into its environment affecting other plants growing in the same habitat (Chou, 2006). Allelochemicals can be produced from any part of the plant (*i.e.*, leaves, flowers, roots, fruits, or stems) and can be released either directly, as in the case of plant root exudates, or indirectly, by leaching of the plants or their residues, as well as by decomposition of plant residues in the surrounding soil (Cheema et al., 2013).

Plant succession is an important process during ecosystem development, especially in ecosystems that are recovering from disturbances such as those in the Horqin Sandy Land. In this region, due to intensive utilization for agriculture and livestock grazing, in combination with the negative effects of climate change (warmer and drier climate) on vegetation, the original grassland was seriously degraded (Zhao et al., 2003). Since about 2000, the Chinese government has adopted many environmental protection projects such as grazing control to restore the grassland ecosystems in the Inner Mongolia Autonomous Region. Thereafter, many degraded grasslands have begun to recover and became denser and more complex communities (Zuo et al., 2009a; Li et al., 2012; Zuo et al., 2012), and both the vegetation cover and the net primary production have increased rapidly (Zhao et al., 2003; Lian et al., 2017). Previous studies have clarified the dynamics of vegetation composition and structure during the restoration process (Zhao et al., 2003; Zhao et al., 2007; Zuo et al., 2009a; Zuo et al., 2009b; Zuo et al., 2012). However, the mechanisms that regulate the dynamics of plant succession during the grassland restoration in this area are still poorly understood (Zhao et al., 2003; Luo et al., 2017).

**Figure 1:**
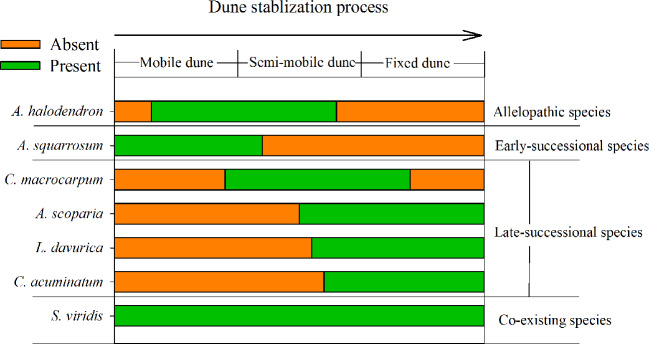
Distribution of the main species during the sand dune stabilization process in the Horqin Sandy Land, northeastern China.

*Artemisia halodendron* is one of the most important subshrubs in the Horqin Sandy Land of northeastern China. It occurs following the appearance of *Agriophyllum squarrosum* in that sandy land (see also **Figure 1**). Due to its deep roots, *A. halodendron* can capture water in the deep soil and therefore exhibits high drought tolerance (Ma et al., 2010). It also has a flexible reproduction strategy that includes both sexual and vegetative propagation (Zhao et al., 2003; Li et al., 2005) and exhibits high tolerance of abrasion by windblown particles and burial by sand (Liu et al., 2008; Huang et al., 2011). Furthermore, its very small seeds facilitate wind dispersal and expansion of its range (Li et al., 2005; Ma et al., 2010).

**Figure 2:**
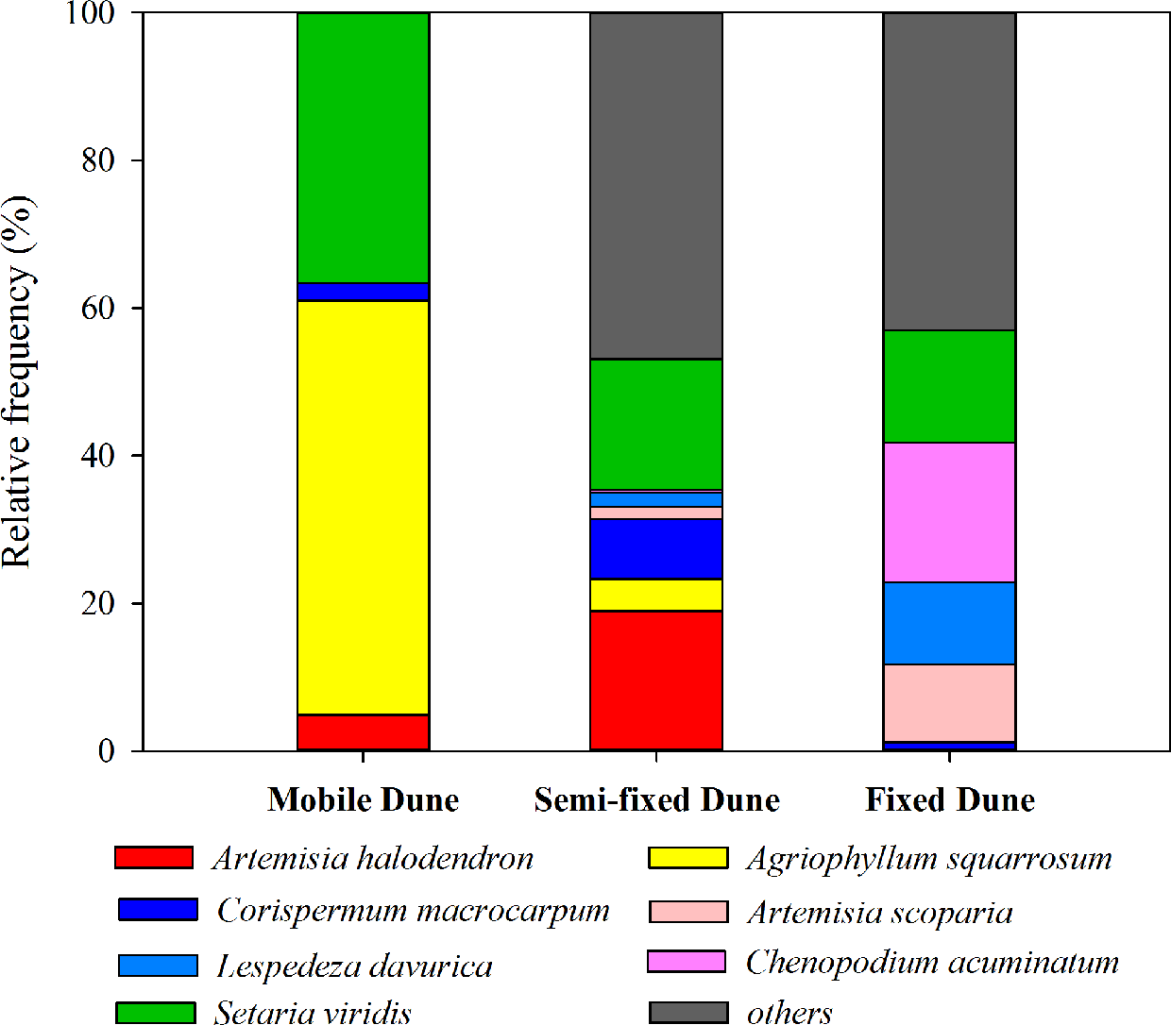
Relative frequency (%) of the main species at three successional stages during the sand dune stabilization in the Horqin Sandy Land, northeastern China.

The plant succession dynamic of dune stabilization has been described in many studies (Zhao et al., 2003; Zuo et al., 2009a; Zuo et al., 2009b). As the mobile dunes become semi-mobile, the density of *A. halodendron* increases sharply, while the density of *A. squarrosum* decreases sharply until the species disappears (see also **Figures 1** and **2**). During this process, the community plant species’ richness increases and several species (*e.g. Artemisia scoparia* Waldst. & Kit, *Lespedeza davurica* (Laxm.) Schindl., *Chenopodium acuminatum* Willd., *Corispermum macrocarpum* Bunge) become established (see also **Figures 1** and **2**). By the time the dunes become fixed, *A. halodendron* disappears completely (see also **Figures 1** and **2**). The mechanisms underlying this species replacement have, however, not yet been studied under natural conditions.

Our previous study (Luo et al., 2017) revealed that the foliage litter of *A. halodendron* had strong allelopathic effects on germination of the soil seed bank and on the seedling growth. However, we still do not know whether not only foliage litter but also stem and root litter of *A. halodendron* have allelopathic effects on other species and whether the intensity of allelopathic effects varies with both litter types (foliage, stem, roots) of *A. halodendron* and the identity of coexisting species. Therefore, we carried out a seed germination experiment to determine the allelopathic effects of three litter types of *A. halodendron* (roots, foliage, and stems) on seed germination of six plant species that progressively occur along a successional gradient in the semi-arid grasslands in the Horqin Sandy Land of northeastern China. We aimed to test our hypotheses that 1) the early-successional species but not the late-successional species respond to the allelopathic effects, and 2) the allelopathic effect increases with increasing concentration of litter extracts, irrespective of litter types.

## Materials and Methods

### Study Site and Plant Species

This study was conducted at the Naiman Desertification Research Station of the Chinese Academy of Sciences (120°43′ E, 42°58′ N; 360 m a.s.l.), which is located in the southwestern part of the Horqin Sandy Land. This desertified area is in the eastern part of China’s Inner Mongolia Autonomous Region. The climate of the region is semi-arid continental monsoon with mean annual precipitation and potential evaporation of 343 and 1935 mm respectively. The mean annual temperature is 6.7°C, with a minimum monthly mean temperature of −12.6°C in January and a maximum of 24.3°C in July. The soil is classified as Cambic Arenosols of sandy origin in the FAO soil classification system (FAO, 2006) and is highly vulnerable to wind erosion.

The landscape is characterized by sand dunes alternating with gently undulating lowlands between the dunes. The plant community is dominated by annual species, with some perennial shrubs and subshrubs (*e.g.*, *A. halodendron*, *Caragana microphylla*, *Salix gordejevii*), and the plant community exhibits high heterogeneity (Zuo et al., 2009a). Based on the natural plant succession dynamics in the Horqin Sandy Land and long-term monitoring of the plant community composition in three categories of habitats (mobile, semi-mobile, and fixed dunes; http://nmd.cern.ac.cn/meta/metaData), six species were chosen to examine their seed germination responses to *A. halodendron*: *A. squarrosum*, *Setaria viridis*, *A. scoparia*, *L. davurica*, *C. acuminatum*, and *C. macrocarpum*. During natural plant succession, *A. squarrosum* occurs first on mobile dunes. Then, as dune stabilization begins, it is replaced by *A. halodendron* on semi-mobile dunes. *Setaria viridis* is widely distributed in northern China, and it often coexists with *A. halodendron*. The other four species are mainly found on semi-mobile or fixed dunes and are therefore late-successional species. During plant species succession, the plant richness and density increased sharply, and *A. halodendron* began to disappear, especially on fixed dunes, where the species were rarely found. **Figures 1** and **2** illustrated the temporal relationships and relative frequency (%) of the main species during three different successional phases of the sand dune stabilization in the Horqin Sandy Land.

### Seed and Litter Sampling

Seeds of these six species were collected at the end of the growing season (late August to October) in 2016 from sand dunes near the station. After air-drying, we preserved all seeds in an unheated storeroom at the station. The storeroom’s winter temperature was as cold as the field, so it should be possible to ignore the effects of seed dormancy due to temperature differences between the storeroom and the field.

At the end of the growing season (mid-September of 2016) when the leaves of *A. halodendron* turned yellow and started falling, three entire plants of *A. halodendron* were excavated then manually separated into leaves, stems, and fine roots (diameter ≤ 2 mm). The root sample was washed up with tap water to remove soil, and then all the three litters (LTs) were air-dried and preserved in the storeroom for further use.

### Litter Extracts Preparation

In early May of 2017, we cut all the three LTs into fragments of 1 to 1.5 cm long, and 100 g of such cut litter of each LT was then steeped in 1 L of distilled water for 24 h. The incubation was conducted in darkness in an incubator held at a temperature of 20°C. We then filtered the extract through a double layer of cheesecloth to remove coarse fragments, followed by filtration through Whatman no. 3 filter paper. Each type of extract was then diluted with distilled water to obtain litter extract with a relative extract concentration (LEC) equivalent of 10, 20, 40, 60, 80, and 100 g·L^−1^. Distilled water was used as the control (0 g·L^−1^). These extracts and the control were then preserved in a refrigerator at 4°C until they could be used (Zhang and Fu, 2010; Samedani et al., 2013).

### Seed Germination Experiment

Healthy-looking seeds with a relatively uniform size were surface-sterilized with 4% (v/v) sodium hypochlorite solution for 2 min followed by immersion in 70% (v/v) ethanol for 2 min. They were then washed three times for 3 min with distilled water. To test the allelopathic effect, we added 5 ml of each LEC (at 0, 10, 20, 40, 60, 80, or 100 g·L^−1^) to sterile 10-cm Petri dishes that contained two sterile Whatman no. 2 filter papers. We used five replicates for each LEC and each species. We placed 30 surface-sterilized seeds of a species in each Petri dish. To avoid contamination and water evaporation, the Petri dishes were sealed with parafilm. Then all Petri dishes were incubated in an incubator. The incubator provides a 14 h photoperiod supplied by white fluorescent lights at a temperature of 25°C. During the nighttime of 10 h, the seeds were kept in darkness at 20°C. This light and temperature regime appears to provide the optimal conditions for germination based on our previous study in the same region (Luo et al., 2017). A total of 630 Petri dishes (3 LTs × 7 LECs × 6 species × 5 replicates) were incubated in the incubator for 21 days. At the end of the germination experiment, we counted the germinated seeds (*i.e.*, seeds that showed both the radicle and a shoot) in each Petri dish and calculated the germination rate in each dish using the following equation:

Germination rate T (%)=[No. of germinated seeds / 30]×100%

where the No. of germinated seeds is the number of germinated seed with fully developed radicle and germ per dish. The 30 is the total number of seeds in each dish.

We defined an allelopathic index (*AI*) to reflect the allelopathic effect of the LTs of *A. halodendron*. *AI* was calculated as follows (Luo et al., 2017):

$$AI=[(T_{x}-T_{C})/T_{C}]\times 100\%$$

where *T_x_* is the germination rate *T* in LEC concentration *x*, and *T_C_* is the germination rate in the control (with a LEC concentration of 0 g·L^−1^). A negative value of *AI* means a negative allelopathic effect of LTs on seed germination, and a positive *AI* value indicates a promoting effect on seed germination.

### Statistical Analysis

Statistical analyses were conducted using SPSS 20.0 software (www.ibm.com/software/analytics/spss/). We used three-way ANOVA to identify the germination responses of the six species to litter extracts concentration (0, 10, 20, 40, 60, 80, and 100 g·L^−1^), litter type (root, foliage, and stem), and their interactions. Due to highly significant effects of species, species × litter type and species × litter extracts’ concentration interaction, we, therefore, used two-way ANOVAs to test the allelopathic effects of the litter type, litter extracts’ concentration (0, 10, 20, 40, 60, 80, and 100 g·L^−1^), and their interaction on seed germination rate for each of the six species, and followed, if significant, by multiple comparisons using the least-significant difference (LSD) test at P < 0.05 level.

## Results

The seed germination rate varied significantly among the six species under LT and LEC treatment (P < 0.001) (**Table 1**). LEC significantly affected the germination (P < 0.001), irrespective of LT (P > 0.05). Different species responded to LT and LEC significantly differently, showing significant species-LT and species-LEC interactions (**Table 1**).

**Table 1:** Results of the three-way ANOVA for the *Artemisia halodendron* litter type (LT) and litter extract concentration (LEC) on seed germination of six common species in a degraded sandy grassland in northeastern China.

Source	*df*	F	*p*
Species (S)	5	928.312	<0.001
Litter type (LT)	2	1.438	ns
Litter extract concentration (LEC)	6	92.832	<0.001
S × LT	10	15.339	<0.001
S × LEC	30	18.023	<0.001
LT × LEC	12	1.114	ns
S × LT × LEC	60	2.339	<0.001

F- and p-values are given. ns, nonsignificant.

**Figure 3:**
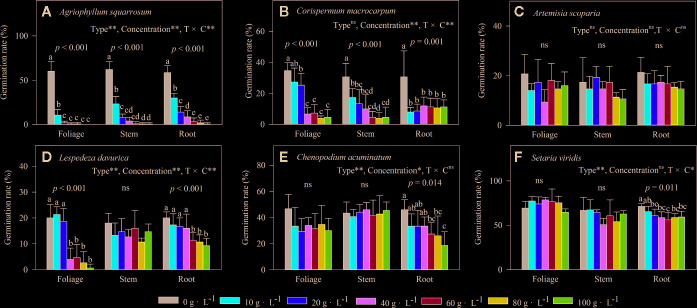
Effects of different concentrations of water extracts from three types of *Artemisia halodendron* litter (foliage, stems, and roots) on seed germination rate of six species **(A–F)** grown in a semi-arid grassland in northeastern China. Values represent means + 1SD. Different lowercase letters (a,b,c,d,e) above the bars indicate significant differences among concentrations (ANOVA followed by LSD test, *P* < 0.05). ns indicates no significant differences among concentrations *P* > 0.05. **(A–F)** indicate the six native species during natural plant succession. **(A)** indicates the early-successional species *A. squarrosum*, **(B–E)** indicate the late-successional species *C. macrocarpum*, *A. scoparia, L. davurica,* and *C. acuminatum*, and **(F)** indicates the co-existing species *S. viridis*.

**Figure 4:**
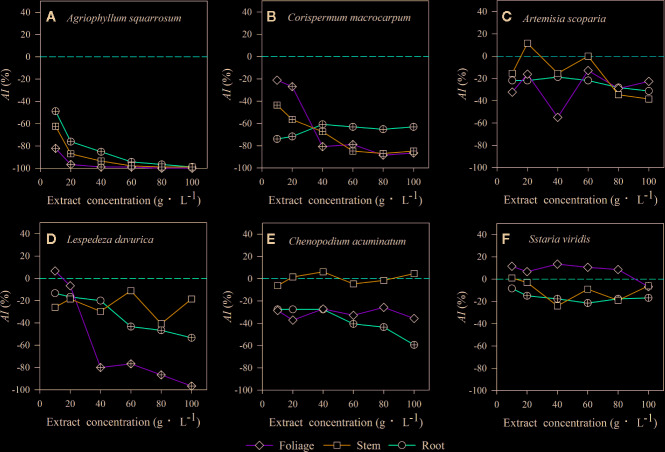
The values of the allelopathic index (*AI*) represent the strength of allelopathic effect of three types of litter extracts from *A. halodendron* on seed germination of 6 native species **(A–F)** grown in a semi-arid grassland in northeastern China. Symbols labeled with a “+” indicate a significant allelopathic effect on seed germination (*P* < 0.05), compared to the control 0-line (*AI* < 0 indicates a negative effect, and *AI* > 0 means a positive effect). **(A–F)** indicate the six native species during natural plant succession. **(A)** indicates the early-successional species *A. squarrosum*, **(B–E)** indicate the late-successional species *C. macrocarpum*, *A. scoparia*, *L. davurica*, and *C. acuminatum*, and **(F)** indicates the co-existing species *S. viridis*.

Extracts of foliage, stem, and roots significantly decreased seed germination of the early-successional species *A. squarrosum* (**Figure 3A**), and this suppression increased with increasing LEC, even though no seeds germinated at the higher LECs (**Figure 3A**). Moreover, the negative effects of LT extracts on seed germination of *A. squarrosum* followed an order of foliage > stems > roots (**Figures 3A** and **4A**), and the *AI* values decreased (more negative) with increasing LEC (−50 to −100%) (**Figure 4A**).

For the relatively late early-successional species, *C*. *macrocarpum*, LTs did not differ in their effects on seed germination (**Figure 3B**). LEC significantly interacted with LT to affect seed germination (**Figure 3B**). At lower LEC of ≤20, the suppression effects had an order of roots > stem > foliage (**Figures 3B** and **4B**), and when the LEC was >40 g·L^−1^, the negative effects of LT extracts on seed germination followed an order of root < stem = foliage (**Figures 3B** and **4B**).

For the three late-successional species, *A. squarrosum* did not respond to LTs and LEC (**Figures 3C** and **4C**). Only root litter extracts had significant negative effects on seed germination of *C. acuminatum* (**Figures 3E** and **4E**), and this suppression increased with increasing root LEC (**Figure 3E**). For *L. davurica*, stem litter extracts had no effects on its seed germination (**Figure 3D**), and also only higher LEC of foliage and root litter extracts significantly suppressed seed germination (**Figures 3D** and **4D**).

For the co-existing species, *S. viridis,* only extracts of root litter significantly decreased its seed germination (**Figure 3F**), and this suppression was weak (less negative *AI* of >−20%) (**Figures 3F** and **4F**).

## Discussion

The present study indicated that the seed germination of the early- (*A. squarrosum*) and early late-successional species (*C*. *macrocarpum*) was significantly suppressed by the extracts of *A. halodendron* litters (**Figures 3A, B** and **4A, B**). Therefore, these two species decreased their relative frequency (**Figure 2**) and finally disappeared with increasing successional stage (**Figure 1**). These findings suggest a significant negative allelopathic effect of *A. halodendron* on other species.

*Agriophyllum squarrosum*, which was the first species that became established on mobile dunes and could be found on mobile dunes only, was strongly inhibited by extracts of all three types of *A. halodendron* litter (**Figure 3a**). Therefore, *A. halodendron* “killed” and replaced *A. squarrosum* quickly after the former became established through allelopathy (**Figures 1** and **2**). This result was also supported by field investigations that *A. squarrosum* and *A. halodendron* distributed separately (Zuo et al., 2009a; Zuo et al., 2012), and *A. squarrosum* seedlings existed never under *A. halodendron* canopy (Zhao et al., 2003). The present study evidenced that litter allelopathy was an important driving force for the replacement of *A. squarrosum* by *A. halodendron*.

The early- (*A. squarrosum*) and the early late-successional species (*C*. *macrocarpum*) were significantly suppressed by the allelopathic effect of *A. halodendron* litters (**Figures 3A, B** and **4A, B**), whereas the late-successional species (*e.g. A. scoparia, C. acuminatum*) were rarely affected by such effects (**Figures 3C, E** and **4C, E**). These findings suggest that the allelopathy of *A. halodendron* seems to accelerate plant succession during the early stage of plant restoration in the Horqin Sandy Land. This agrees with the hypothesis that allelopathy can increase the rate of plant succession (Rice, 1984).

Allelopathy can also inhibit plant succession when early-successional species suppress the establishment or growth of late-successional species (Mallik, 2003; Fernandez et al., 2006). In the present study, seed germination of a late-successional species (*L. davurica*) was generally suppressed by *A. halodendron* litter (**Figures 3D** and **4D**). This may imply that *A. halodendron* allelopathy influences the late succession, at least affects the species composition and structure of the community. This partly supports the statement of previous studies that allelopathy can also suppress or slow successional change (Mallik, 2003; Fernandez et al., 2006; Mudrák and Frouz, 2012).

The *A. halodendron* litter extracts did not affect the seed germination of *A. scoparia* (**Figures 3C** and **4C**), indicating that the pioneer subshrub *A. halodendron* cannot influence seed germination of all late-successional species similarly. The theory of kin recognition suggests that plants that are genetically related tend to decrease their competitive interactions by modifying their morphology, such as reducing root overlap and increasing leaf area to avoid resource competition (Ninkovic, 2003; Dudley and File, 2007; Bhatt et al., 2011). Given the fact that *A. halodendron* and *A. scoparia* belong to the same genus, it’s possible that kin selection by reducing competitive interactions may partially explain the lack of significant allelopathy for *A. scoparia*.

For the co-existing species, *S. viridis*, its seed germination was also significantly negatively affected by the root litter of *A. halodendron* (**Figures 3F** and **4F**), but it exists always in the community (**Figures 1** and **2**). Previous studies demonstrated that *S. viridis* had high tolerance to environmental stresses (Huang et al., 2008). For example, it had much stronger drought resistance than *A. squarrosum* in the Horqin Sandy Land (Huang et al., 2008). It also tolerated competition with the invasive species *Ageratina adenophora*, which threatened the growth and survival of many native species in southern China (Zhou et al., 2006). This tolerance may explain how *S. viridis* does resist allelopathy and competition in that community.

The plant succession in the study region is influenced by many factors such as plant propagation materials (Luo et al., 2017), soil water, and nutrients (Zhao et al., 2011), and also litter allelopathy (Zhang et al., 2015). However, the vegetation community composition varies greatly with the distance from *A. halodendron* plant (Zhao et al., 2007), although there are hardly differences in soil nutrients and water conditions in the root layer (0–40 cm) within that distance in Horqin sandy land (Zhao et al., 2003). This suggests that the allelopathy of *A. halodendron* plays an important role in determining the survival and development of its neighboring plant species. The allelochemicals may be produced in many plant parts during plant growth and development to exert multiple ecological effects and thus to affect the co-existing plants (Luo et al., 2012). In natural conditions in Horqin sandy land, plant seeds are mainly distributed in the surface soil (Zhao et al., 2007), and thus, the allelopathy of *A. halodendron* litters may mainly affect seed germination, while the allelopathy of *A. halodendron* root exudates may mainly influence the establishment, growth, and survival of its neighboring plants.

The result of this study evidenced that the effect of litter extracts of *A. halodendron* on seed germination differed among litter types (**Figure 3**); this may be a result of different chemical compositions and contents existing in various litter types. Although we did not determine the chemical composition and content of the litter extracts of each litter type in this study, a previous study showed that there are significant differences in the chemical quality/quantity and decomposition process among litter types of *A. halodendron* (Li Y. et al., 2016). The seed germination of *S. viridies* was only weakly inhibited by the extracts of the root litter of *A. halodendron* (**Figures 3F** and **4F**), and thus, *S. viridies* and *A. halodendron* can co-exist successfully in the long-term in nature (Zuo et al., 2009a; Zuo et al., 2012). For the latter successional species, such as *C. acuminatum*, its seed germination was only inhibited by the extracts of root litter of *A. halodendron* (**Figures 3E** and **4E**), but in the field, the growth of *C. acuminatum* is strongly affected by *A. halodendron,* probably due to root connection and belowground allelopathy. Here we only evidenced that litters of *A. halodendron* can affect the seed germination through allelopathy; further studies are needed to investigate the aboveground and especially the belowground impacts of *A. halodendron* on the growth and replacement of plant species along vegetation succession in the sandy regions. Moreover, future studies should also take the effective components of *A. halodendron* litters, their physiological properties and effects into account.

## Conclusions

In line with our hypotheses, the present study indicated that the early-successional species rather than the late-successional species were negatively affected by the allelopathic effects of *A. halodendron*, and that the allelopathic effects on seed germination increase with increasing concentration of litter extracts, irrespective of litter types. Our study represented the first evidence that allelopathy may be a potentially important driving force during the early-successional stage and dune stabilization in the Horqin Sandy Land. A limitation of the present study is that we did not check whether other species (*e.g.* the late-successional species) have also negative allelopathic effects on *A. halodendron*, leading to the disappearance of *A. halodendron* in the late-successional stages (**Figures 1** and **2**). On the other hand, the disappearance of *A. halodendron* in the late-successional stages may also be a result of changed growth environment including dune stabilization and plant competition. Therefore, further studies are needed to better understand the successional process and thus to promote the vegetation restoration in that sandy dune region.

## Data Availability Statement

All datasets generated for this study are included in the article/supplementary material.

## Author Contributions

YLuo and ZD designed the study. YLuo and ZY conducted the field trial. YLuo and ZY performed the laboratory analysis. YLuo, ZD, and M-HL were responsible for the statistical analyses. YLuo, ZD, ZY, XZ, and YLi wrote the original draft paper. YLuo, ZD, HJ, YY, and M-HL critically reviewed and edit the preliminary draft paper. All authors contributed to the article and approved the submitted version.

## Funding

This research was funded by the National Key Research and Development Program of China (2017YFA0604803, 2016YFC0500907), Ecological and Security Key laboratory of Sichuan Province (ESP1708), National Natural Science Foundation of China (No. 31500369, 41807525, 41501227), and Sichuan Science and Technology Program (2018JY0086), China West Normal University Program (17E038), The Strategic Priority Research Program of Chinese Academy of Sciences (XDA24020304).

## Conflict of Interest

The authors declare that the research was conducted in the absence of any commercial or financial relationships that could be construed as a potential conflict of interest.
